# Changing Face of Ham Green Hospital

**Published:** 1987-02

**Authors:** Dennis W. Wright

**Affiliations:** Physician, Occupational Health Department, Assist, Physician, Ham Green Hospital, Pill


### The changing face of Ham Green Hospital

**Dennis W. Wright MB, ChB.**
Physician, Occupational Health Department, Assist, Physician, Ham Green Hospital, Pill.

The Hospital opened on 12 July 1899 and replaced a hulk anchored in the river opposite Avonmouth. The material for its building and then its patient scame by boat from the City of Bristol and the city boundary was extended by 700 acres. Initially there were 150 beds with a medical superintendent in charge. Dr B A I Peters came in 1907 with Miss Garden as Matron and remained for over 40 years. He saw the hospital grow to 350 beds and 250 for tuberculosis with a further 100 beds at Charterhouse on the Mendips. There was no bacteriology or x-ray service and no telephone. Wards summoned the night sister by ringing a large bell. With the introduction of the National Health Service in 1948, Dr Peters retired and was replaced by Dr James Macrae as Consultant General Physician, contracted to live at Ham Green, a fever hospital surrounded by a high wooden fence and visitors were not encouraged.

Dr Macrae developed a bacteriology service and the hospital started to respond to change with a Management Committee. Antibiotics started to have an effect on recovery and survival.

In 1953 Chest Physicians, Dr Robert Craig and Dr Allan Roberts, took charge of the sanatorium, later joined by Dr's Hoffman, Davies and Iles. The wooden fence disappeared and the fever hospital received its first visitors.

Polymyelitis spread through the Country and artificial respiration became a necessary part of its treatment. At this time (1953) Dr Ronald Walley joined Dr Macrae and they eventually, in 1958, developed one of the first intensive care units in Britain.

This unit cared for 186 cases of severe tetanus, chronic bronchitis and a wide range of muscle paralysis patients.

With the wider use of immunisation the number of cases of tetanus fell and nowadays those that are seen are usually treated by anaesthetists in intensive care units. The use of BCG and the introduction of anti-tuberculous drugs has reduced the need for TB beds so that now Ham Green, which is the only hospital in the area for the medical treatment of TB, has less than a dozen in-patients at any one time. In 1959 the Hospital became mainly acute and acquired more medical staff, both senior and junior (Dr Cates was one of the physicians at the time). Due to the foresightedness of Mr Christopher Hancock, Southmead Group Secretary, an artificial kidney was acquired and was first used at Ham Green and the service gradually built up and improvements in both technique and equipment were developed. With the introduction of the specialty of Nephrology this work was later transferred to Southmead Hospital. Dr Stuart Parker joined about this time and was concerned with both this work and other in the intensive care unit. In the 1960's Ham Green performed 15,000 operations for tonsils and adenoids and reduced the waiting list considerably.

Ham Green had the first purpose built unit in the country for the young chronic sick, developed by Dr Francis Page (assisted by Dr Ian Hawkins, a local GP). When Dr Page retired he was replaced by Dr Iain Ferguson.

In the 1970's a coronary care unit was developed on a general medical ward, supervised by Dr D W Barritt.

The Hospital had one of the first comprehensive occupational health services in the country which provided a full service for all grades of staff.

However, in 1984 the service was centralised at Southmead Hospital, since when the Occupational Health Nurse is only available at Ham Green on two half days a week. At other times staff are seen on a ward or referred to Southmead.

In 1972 a Unit for Research and investigation and treatment of urinary incontinence was set up under the leadership of Mr Roger Feneley. Since then it has become well known throughout this Country and abroad and members of the research team have read many papers about their work.

During the last 10 years the number of chest physicians has been reduced from originally three (plus two assistants) until now when we have only one, Dr John Harvey who is also a general physician. Dr Nick Hoffman left us in August 1986 to go to the General Hospital, taking with him some of the staff and patients, though he still has an outpatient clinic at Ham Green. Dr Ian Bailey, who had a wide range of interests including general medicine at Ham Green, went back to Southmead in 1985 and was not replaced.

During the last 18 years a comprehensive geriatric service has been developed by Dr Geoffrey Burston and colleagues, later joined by Dr William Calwell. This was followed by a Psychogeriatric service under the leadership of Dr Ihsanul Mian and more recently Dr Richard Hawley. Both units have Day Hospital facilities and serve the local community.

The beds originally occupied by TB patients were first of all used for gynaecology and urology and when it became obvious that there was a need for more surgical beds for patients from the Weston area while their Hospital was being built, a new theatre block was built and the Weston surgeons came up to operate. Now the new hospital has opened and they have left us, with their patients, and the beds are presently being used by the urologists while essential work is being done at Southmead. It is expected that urology and gynaecology will return to Southmead in April 1987 and the wards will again be empty and available for other use.

In 1978 a unit was set up to treat exotic diseases (viral haemorrhagic fevers including Lassa) using a Trexler tent. A few suspect cases have been nursed in the unit. In 1985 Dr Glover, who replaced Dr Walley in 1982, travelled to Sierra Leone to bring back a young girl with Lassa Fever and she was nursed back to health in the Unit. Dr Stuart Glover is keeping Ham Green in the public eye by his regular appearances on radio and television and in the newspapers and because of his interest in the relatively new disease AIDS.

The various reorganisations in the Health Service have brought many changes and we wait to see their full effects.

What of its eventual future? Will it remain, albeit further reduced in size and perhaps changed in function?

The projected 10 year plan for the Southmead District recommends that all acute services will eventually leave the Ham Green site and go to Southmead Hospital. What will remain has not yet, I understand, been finally decided, though it is likely that the geriatric services and services for the elderly mentally infirm will remain, at least for some time. The number of beds in use has reduced from 400 in the 1950's to 270 today.

Ham Green is on a very attractive, flat, though scat-

*(continued on page 16)*

*(Changing Face of Ham Green Hospital continued from page 14)*

tered, site and has good access to the Motorway (less than a mile away). At present it is designated Green Belt so cannot be sold for housing when it might have fetched a lot of money (£3 million has been suggested). Meanwhile, it will continue to function and provide a service and all who work in it hope and expect this will be for several years to come.

---

**ACKNOWLEDGEMENT**

I would like to thank Dr James Macrae for providing me with much of the information and help in compiling this report.

